# Expression of Human DNAJ (Heat Shock Protein-40) B3 in Humanized *UDP-glucuronosyltransferase 1* Mice

**DOI:** 10.3390/ijms160714997

**Published:** 2015-07-02

**Authors:** Ryo Mitsugi, Tomoo Itoh, Ryoichi Fujiwara

**Affiliations:** Department of Pharmaceutics, School of Pharmacy, Kitasato University, Tokyo 108-8641, Japan; E-Mails: pl12032@st2.pharm.kitasato-u.ac.jp (R.M.); itoht@pharm.kitasato-u.ac.jp (T.I.)

**Keywords:** heat shock protein, DNAJB3, humanized animal model, obesity

## Abstract

The human *DNAJB3* gene encodes a DNAJ (Heat shock protein 40; Hsp40) homolog, subfamily B, member 3 chaperone protein (DNAJB3), which can be down-regulated in disease conditions, as observed in decreased expression of DNAJB3 mRNA in peripheral blood mononuclear cells (PBMC) of obese patients. Recently, humanized *UDP-glucuronosyltransferase* (*UGT*) *1* mice (*hUGT1* mice) were developed, in which the introduced human *UGT1* gene contained a gene encoding human DNAJB3. In the present study, we analyzed the expression of human DNAJB3 mRNA in *hUGT1* mice. Among the examined tissues, the testis had the highest expression of human DNAJB3 mRNA, while the lowest expression was observed in the liver. We found that the pattern of tissue-specific expression of mouse Dnajb3 in *hUGT1* mice was very similar to that of human DNAJB3. We further demonstrated that the expression of human DNAJB3 in the liver was significantly reduced in high-fat-diet-fed *hUGT1* mice compared to the expression level in the control mice, indicating that the expression of human DNAJB3 in *hUGT1* mice could be similarly regulated in disease conditions such as obesity. Humanized *UGT1* mice might therefore be useful to investigate the physiological role of human DNAJB3 *in vivo*.

## 1. Introduction

Heat shock proteins (HSPs) were originally defined as a group of proteins induced by heat shock [1]. HSPs are now widely recognized as cellular proteins that can be induced under various stress conditions such as hypoxia and virus infection [2,3]. While the basic function of HSP includes regulation of cell growth, apoptosis, and protein homeostasis [2], recent studies have shown that various HSPs are highly expressed in cancer cells, possibly to promote or suppress their proliferation [4]. Certain HSPs were significantly up-regulated in disease conditions [5], suggesting that HSPs can be markers to diagnose various diseases.

DNAJ, also known as HSP-40, is a chaperone protein. In humans, 41 DNAJ member proteins are classified into three families, DNAJA, DNAJB, and DNAJC, which contain 6, 12, and 23 members, respectively. Among DNAJB family proteins, DNAJB1 is the most extensively studied member in mammalian cells. It was demonstrated that Dnajb1 mRNA was exclusively expressed in mouse testis and its protein was expressed in the cytoplasm of cells [6]. As it was found that DNAJB1 interacts with HSPA1A and HSPA8, which are HSP70 members, in luciferase refolding *in vitro* and in living cells [7,8], the J domain of the DNAJ family members is important when they bind to their partner HSP70s [9,10]. Details on the function of DNAJB members as chaperone proteins have been reviewed by Qiu *et al.* and Vos *et al.* [11,12]. While DNAJB1 is not induced by heat shock [6], a recent study demonstrated that DNAJB3 was down-regulated in disease conditions, as decreased expression of DNAJB3 mRNA was observed in peripheral blood mononuclear cells (PBMC) of obese patients. It was further demonstrated that the mRNA expression of DNAJB3 was restored by physical exercise, suggesting that DNAJB3 could potentially play a protective role against obesity [13]. However, little is known about the gene regulation and function of DNAJB3 or the potential usefulness of DNAJB3 as a marker gene for disease susceptibility.

**Figure 1:**
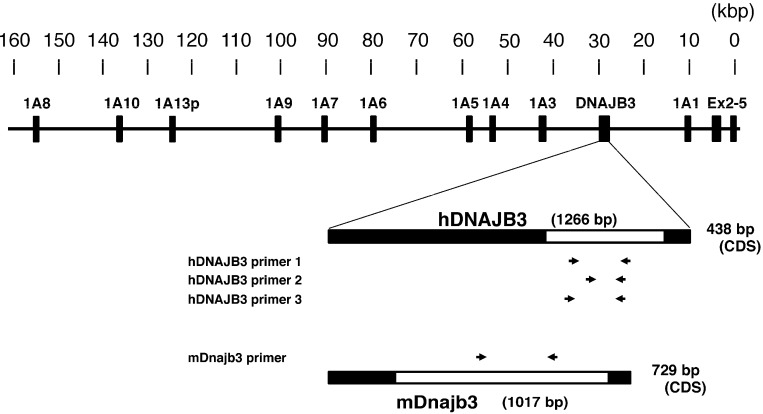
Human DNAJ family homolog, subfamily B, member 3 (*DNAJB3*) locus on the human *UDP-glucuronosyltransferase* (*UGT*) *1* (*UGT1*) gene. Human *DNAJB3* gene is located between the UGT1A1 exon1 and the UGT1A3 exon1. The lengths of human *DNAJB3* and mouse *Dnajb3* gene are 1266 and 1017 bp (bars) and the lengths of cording sequence are 438 and 729 bp (white bars), respectively. Mouse *Dnajb3* is located in the mouse *Ugt1* locus. Primer binding areas are shown with arrows.

Transgenic and humanized mice are frequently used to understand the regulation or function of human genes *in vivo*. UDP-glucuronosyltransferase (UGT) is an important drug-metabolizing enzyme that catalyzes a transfer of glucuronic acid from a co-substrate, UDP-glucuronic acid, to substrates [14]. Previously, to understand human-specific neonatal jaundice, and to overcome species difference of the glucuronidation activities between humans and mice, we established humanized *UGT1* (*hUGT1*) mice in which the original *Ugt1* locus was disrupted and replaced with the human *UGT1* locus [15,16]. While the *hUGT1* mice were remarkably useful in the fields of drug metabolism and pharmacokinetics, it was further discovered that the human *UGT1* transgene, which was introduced in the *hUGT1* mice, contained a gene encoding DNAJB3 (Figure 1).

In the present study, we analyzed the expression of human DNAJB3 mRNA in *hUGT1* mice. The expression pattern of human DNAJB3 in various tissues was determined and compared to that of mouse Dnajb3 in *hUGT1* mice. We further examined the effects of high-fat diet (HFD) or a UGT inducer on the expression of DNAJB3.

## 2. Results

### 2.1. Specificity of the Primers

**Figure 2:**
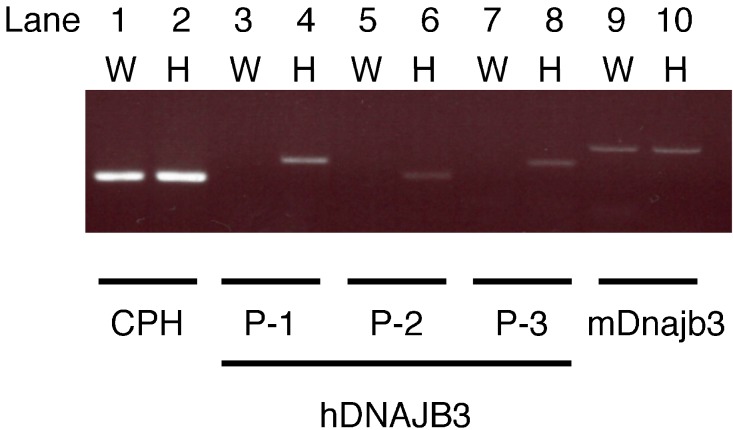
Electrophoretic analysis of the RT-PCR products. Total RNA of wild-type mouse liver (W) and humanized *UDP-glucuronosyltransferase 1* (*hUGT1*) mouse liver (H) were used for the RT-PCR analysis using primer pairs for human DNAJB3 and mouse Dnajb3. Cyclophilin B (CPH) was used as a loading control.

Human DNAJB3 and mouse Dnajb3 share 75% amino acid sequence similarity. In the present study, we designed three sets of primers, hDNAJB3-1, -2, and -3, specific to human DNAJB3. In wild-type mice, RT-PCR using these primers did not produce any bands (Figure 2, lane 3, 5, and 7), indicating that the human DNAJB3 primers did not react with mouse Dnajb3. In contrast, specific bands were obtained in the RT-PCR using these primers in *hUGT1* mice (Figure 2, lane 4, 6, and 8), confirming that human DNAJB3 mRNA was expressed in *hUGT1* mice and that primers specific to human DNAJB3 could amplify the corresponding fragments. Specific bands were obtained in the RT-PCR analysis using primers specific to mouse Dnajb3 in wild-type mice and *hUGT1* mice (Figure 2, lane 9 and 10). This indicated that mouse Dnajb3 mRNA was similarly expressed in wild-type mice and *hUGT1* mice. Among the three sets of human DNAJB3 primers, hDNAJB3-1 primers were used for the following experiments.

### 2.2. Tissue-Specific Expression of Human DNAJ Family Homolog, Subfamily B, Member 3 (DNAJB3) in Humanized UDP-Glucuronosyltransferase 1 (hUGT1) Mice

**Figure 3:**
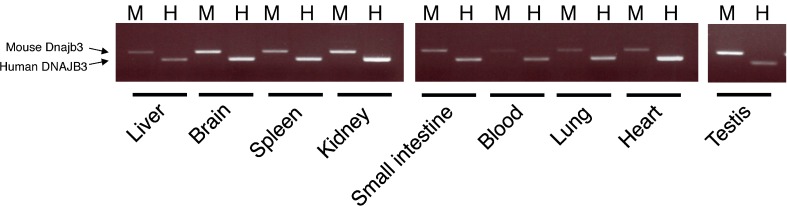
Expression of human DNAJB3 and mouse Dnajb3 mRNA in *hUGT1* mice. The expressions of human DNAJB3 (H) and mouse Dnajb3 (M) were analyzed by RT-PCR. Total RNA was isolated from liver, blain, spleen, kidney, small intestine, blood, lung, heart and testis of *hUGT1* mice.

In 2004, Meccariello *et al.* demonstrated that mRNA of Dnajb3, which was also known as mouse sperm cell-specific Dnaj first homologue (Msj-1), was abundant in mouse and frog testis [17]. Recently, it was demonstrated that human DNAJB3 mRNA was expressed in PBMC [13]. However, expression patterns of human DNAJB3 and mouse Dnajb3 are not fully understood. In the present study, the liver, small and large intestine, kidneys, lungs, heart, blood, and testis were isolated from *hUGT1* mice and the expression of human DNAJB3 and mouse Dnajb3 mRNA was determined by RT-PCR. In the liver, both human DNAJB3 and mouse Dnajb3 were slightly expressed (Figure 3).

**Figure 4:**
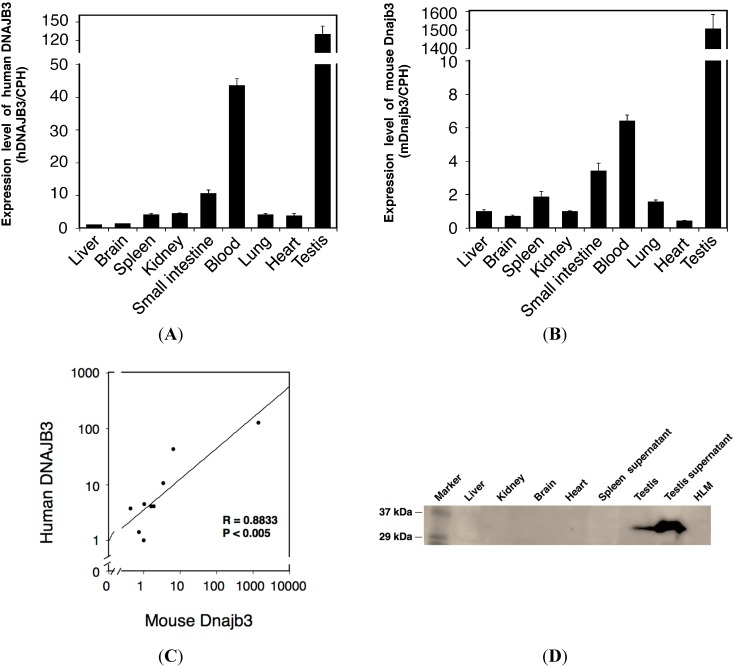
Quantitative analysis of the expressions of human DNAJB3 and mouse Dnajb3 mRNA in *hUGT1* mice. Expressions of human DNAJB3 (**A**) and mouse Dnajb3 (**B**) were quantitatively analyzed by Q-RT-PCR. Expression levels were normalized with the expression of Cyclophilin B (CPH) and were compared with the expression level in the liver; (**C**) Correlation between mouse Dnajb3 and human DNAJB3 mRNA expression in *hUGT1* mouse tissues was analyzed. The correlation analysis was carried out using the Spearman rank method. Regression line was shown; Western blotting was carried out to detect DNAJB3 protein expression in *hUGT1* mice (**D**). Liver (127 µg), kidney (36 µg), brain (54 µg), heart (50 µg), and testis (50 µg) homogenates, spleen (100 µg) and testis (500 µg) supernatant, and HLM (100 µg) were subjected to the immunoblotting analysis. R, correlation coefficient; *p*, value of significance; HLM, human liver microsomes.

To quantitatively determine the expression of human DNAJB3 and mouse Dnajb3 mRNA in those tissues, we further conducted a Q-RT-PCR analysis. As observed in the semi-quantitative PCR analysis, the Q-RT-PCR analysis showed the lowest and highest expressions of human DNAJB3 in the liver and testis (Figure 4A). It was revealed that human DNAJB3 mRNA was relatively highly expressed in the lung, spleen, blood, small intestine, heart, and kidney compared to the expression level in the liver. The highest expression of mouse Dnajb3 was also observed in the testis. While the liver, brain, heart, and kidney showed slight expressions of mouse Dnajb3, other tissues such as the lungs, spleen, blood, and small intestine showed relatively higher expressions of mouse Dnajb3 (Figure 4B).

To understand whether human DNAJB3 and mouse Dnajb3 were similarly expressed in the *hUGT1* mouse tissues, we conducted a correlation analysis. The expression levels of human DNAJB3 in the tissues were plotted against the expression levels of mouse Dnajb3 in the same tissues (Figure 4C). The statistical study revealed that the correlation of the expression of human and mouse DNAJB3 (Dnajb3) was statistically significant (*p* < 0.05), suggesting that the pattern of tissue-specific expression of human DNABJ3 is very similar to that of mouse Dnajb3 in *hUGT1* mice. As the testes had the highest expression of DNAJB3, we observed a strong band in the testes in the Western blot analysis (Figure 4D).

### 2.3. Effects of a High-Fat Diet on the Expression of Human DNAJB3 in hUGT1 Mice

**Figure 5:**
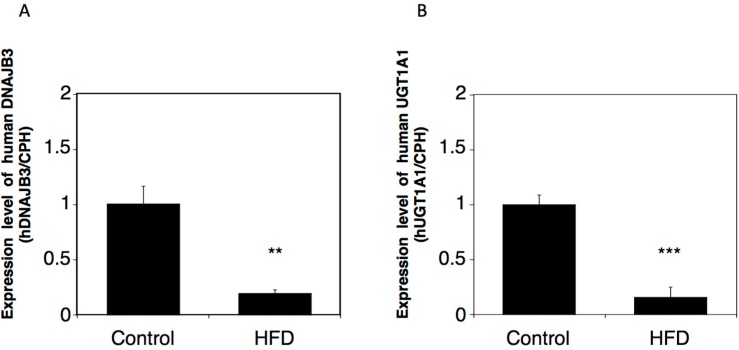
Effect of HFD on the mRNA expression of human DNAJB3 and UGT1A1 in *hUGT1* mice. Total RNA was extracted from the liver of control and HDF-fed *hUGT1* mice. Human DNAJB3 (**A**) and UGT1A1 (**B**) mRNAs were quantitatively analyzed by Q-RT-PCR in male *hUGT1* mice. Expression levels were normalized with CPH and were compared with the expression in the control mice. HFD, high-fat diets. ** *p* < 0.01 and *** *p* < 0.001.

In humans, a decreased expression of DNAJB3 mRNA was observed in obese patients. To examine whether a similar regulation of the expression of DNAJB3 could be observed in *hUGT1* mice, we examined the expression of human DNAJB3 in control and HFD-fed *hUGT1* mice. Obesity model *hUGT1* mice were established by feeding the mice with HFD for two months. In the male mice, the DNAJB3 mRNA level in the HFD-fed group was 5-fold lower than the level in the control group (Figure 5A). In the female mice, the DNAJB3 mRNA level in the HFD-fed group was 3-fold lower than the level in the control group (data not shown). These findings indicate the possibility that the expression of DNAJB3 in *hUGT1* mice can be similarly reduced by human disease conditions such as obesity. In the male HFD-fed *hUGT1* mice, UGT1A1 expression was significantly decreased in the liver (Figure 5B).

### 2.4. Effects of a UGT Inducer on the Expression of Human DNAJB3 in hUGT1 Mice

**Figure 6:**
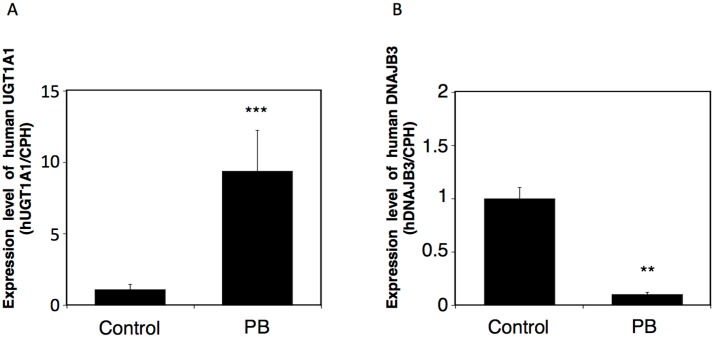
Effects of phenobarbital on the mRNA expressions of UGT1A1 and human DNAJB3 in *hUGT1* mice. The *hUGT1* mice were orally treated with phenobarbital (100 mg/kg/day) for three days. Twenty-four hours after the last treatment, the liver was isolated and the total RNA was extracted from the tissue. The expression levels of UGT1A1 (**A**) and human DNAJB3 (**B**) mRNA were quantitatively analyzed by Q-RT-PCR. Expression levels were normalized with the CPH expression and were compared with the level in control mice. PB, phenobarbital. ** *p* < 0.01 and *** *p* < 0.001.

The *UGT1* gene produces nine functional UGT1A isoforms, UGT1A1, UGT1A3, UGT1A4, UGT1A5, UGT1A6, UGT1A7, UGT1A8, UGT1A9, and UGT1A10, by exon sharing. Phenobarbital is an indirect activator of a nuclear receptor, constitutive-active/androgen receptor (CAR). It has been shown that phenobarbital can induce most of the UGT1 members in the *hUGT1* mice [18]. Since the *DNAJB3* gene is a part of the *UGT1* gene (Figure 1), we hypothesized that the transcription of DNAJB3 would be interfered with by a significant induction of the *UGT1* gene. Phenobarbital induced UGT1A1 six-fold in the liver (Figure 6A), which is in agreement with a previous report [18]. In contrast, the expression of DNAJB3 mRNA was 10-fold lower in the phenobarbital-treated *hUGT1* mice compared to the control mice (Figure 6B). These findings indicate that the transcription of DNAJB3 can be interfered with by *UGT* inducers.

## 3. Discussion

The *UGT1* gene encodes various UGT1A family enzymes. UGTs are known as drug-metabolizing enzymes, and most of the UGT1A proteins can metabolize clinically used drugs such as ibuprofen, furosemide, imipramine, and propofol. Meanwhile, they are also involved in metabolism of endogenous substances such as bilirubin, estradiol, and serotonin. UGT1A1-catalyzed glucuronidation is a rate-limiting reaction in bilirubin metabolism in humans; therefore, genetic polymorphism in the *UGT1A1* gene can cause hyperbilirubinemia. Although human neonates physiologically develop mild hyperbilirubinemia, called jaundice, such a phenotype is not observed in other mammals such as mice or rats due to their extremely high UGT1A1 activity during the neonatal period. Humanized *UGT1* mice were previously developed to understand the molecular mechanism of neonatal jaundice in humans. It was found that reduced expression of gastrointestinal UGT1A1 was the cause of breast milk-induced neonatal jaundice. Humanized *UGT1* mice were also useful to overcome the species difference in glucuronidation of drugs between humans and mice [19,20].

Chaperone proteins promote folding, assembly, and translocation into organelles of newly synthesized proteins. The human *DNAJB3* gene encodes a DNAJ (Hsp40) homolog, subfamily B, member 3 chaperone protein (DNAJB3). DNAJB3, which is also known as Msj-1 in mice, was originally identified as being highly expressed in the testes [21]. Subsequently performed studies showed that DNAJB3 was also expressed in other tissues such as the spinal cord, central nervous system, sperm cells [22,23], and brain. A variety of functions of DNAJB3 have been reported. In mice, for example, the Dnajb3 protein exhibits an important role in acrosomogenesis [24]. Due to a lack of key amino acid residues, the human DNAJB3 was reported to be inactive as a chaperone protein [25]. Meanwhile, a recent study demonstrated that human DNAJB3 was potent enough to form protein-interactions with HSP-72 and stress kinases such as c-Jun NH_2_ terminal kinase (JNK) and inhibitor of κB kinase-β (IKKβ) [13]. Furthermore, it was demonstrated that the expression of human DNAJB3 was dramatically suppressed by ER stress. However, the functional importance of human DNAJB3 *in vivo* remains to be determined, as most of these data were obtained by *in vitro* studies. Interestingly, the *UGT1* gene introduced in the *hUGT1* mice contained the *DNAJB3* gene between the exon 1s for UGT1A3 and UGT1A4 (Figure 1). Expression patterns of human DNAJB3 in *hUGT1* mice were very similar to those of mouse Dnajb3 (Figure 4C). Our humanized *UGT1* mice, therefore, might be useful to investigate the physiological role of human DNAJB3 *in vivo*.

Biomarkers are used to measure exposure to a toxic agent, to detect severity of any toxic response, and also to predict disease conditions. In addition to the role of DNAJB3 as a chaperone protein and its ability to bind other proteins, it was proposed that DNAJB3 could be a biomarker to detect obese conditions [13]. In the present study, we demonstrated that the expression of human DNAJB3 was significantly reduced in the HFD-fed *hUGT1* mice (Figure 5), indicating that human DNAJB3 is similarly regulated in *hUGT1* mice as observed in humans. Therefore, in addition to the potential usefulness of *hUGT1* mice to investigate the physiological role of human DNAJB3, our *hUGT1* mice could also be used to investigate how reliable human DNAJB3 is as a biomarker to detect obese conditions. Not only phenobarbital, but also other factors such as endogenous hormones and inflammation can regulate the expression of UGTs by affecting the function of transcriptional factors like Pregnane X receptor (PXR), Aryl hydrocarbon receptor (AhR), Peroxisome proliferator-activated receptors (PPARs), and nuclear factor-κB (NF-κB). It was demonstrated that the function of PPARs was modulated in obesity [26], suggesting that those might be the factors regulating the expression of DNAJB3 in obese patients.

It has been reported that hepatic UGTs are highly induced by enzyme inducers in mice [27]; therefore, we mainly examined the impact of phenobarbital on the expression of UGTs and DNAJB3 in the tissue. It will be important to investigate the effect of phenobarbital and a high-fat diet on the expression of UGTs and DNAJB3 in other tissues such as blood (PBMC), adipose tissue, and skeletal muscle in future studies. Identification of the function of DNAJB3 and its importance in obesity needs to be addressed in the future.

## 4. Experimental Section

### 4.1. Chemicals and Reagents

Phenobarbital was purchased from Wako Pure Chemical (Osaka, Japan). Primers were commercially synthesized at Life Technologies (Carlsbad, CA, USA). All other chemicals and solvents were of analytical grade or the highest grade commercially available.

### 4.2. Animals and Treatments

Tg(UGT1A1*28)Ugt1^−/−^ (*hUGT1*) mice were developed previously in a C57BL/6 background [15]. All animals received food and water *ad libitum*, and mouse handling and experimental procedures were conducted in accordance with our animal care protocol, which was previously approved by Kitasato University (Kitasato University Animal Care and Use Committee #A13-2, approved on 30 April 2014 and 1 May 2015). For tissue collections, mice were anesthetized by diethyl ether inhalation, and the liver was perfused with ice-cold 1.15% KCl. The tissues were isolated and rinsed in cold 1.15% KCl and stored at −80 °C. Humanized *UGT1* mice were orally treated with phenobarbital (100 mg/kg/day) for three days. Twenty-four hours after treatment, the mice were anesthetized and tissues were isolated. The tissues were also isolated from *hUGT1* mice fed with a high-fat diet for two months.

### 4.3. Reverse-Transcription Polymerase Chain Reaction (RT-PCR) and Quantitative (Q)-RT-PCR

Total RNA was extracted from tissues with Trizol reagent (Invitrogen, Carlsbad, CA, USA). The cDNA was synthesized from total RNA using ReverTra Ace (TOYOBO, Osaka, Japan) according to the manufacturer’s protocol. The following PCR primers and condition were used to detect human DNAJB3 and mouse Dnajb3: hDNAJB3-sense primer 1, 5′-GAGGCCTACGAGGTGTTGTC-3′ and antisense primer 1, 5′-CGGGTTTCCCAAGAGGTCAA-3′; hDNAJB3-sense primer 2, 5′-GTTGTCGGACGCCAAGAAAC-3′ and antisense primer 2, 5′-GAACTCCCTGAAGACGTCGG-3′; hDNAJB3-sense primer 3, 5′-TGTTGTCGGACGCCAAGAAA-3′ and antisense primer 3, 5′-TATTCTCCAGCGGGTTTCCC-3′; mDnajb3-sense primer, 5′-GCAGGCCTACGAGGTCTTAT-3′ and antisense primer, 5′-TAGAGAAGGGTACAGCCCCT-3′; 94 °C for 30 s, 58 °C for 30 s, and 72 °C for 30 s for 30 cycles.

Q-RT-PCR was performed with THUNDERBIRD SYBR qPCR Mix (TOYOBO), and the reactions were run in a CFX96™ Real-Time PCR Detection System (BioRad, Hercules, CA, USA). Primers for human UGT1A1 and mouse cyclophilin B (CPH) were developed previously [16]. Expression of CPH mRNA was used as an internal control for cDNA quantity and quality. The following PCR conditions were used: Denaturation at 95 °C for 5 s, annealing at 60 °C for 15 s, and extension at 72 °C for 30 s for 40 cycles.

### 4.4. Western Blotting Analysis

Tissue homogenates were prepared. Tissue homogenates were further centrifuged at 600 g for 10 min to obtain their supernatants. Tissue homogenates and supernatants were subjected to neutral polyacrylamide gel electrophoresis (NuPAGE) 4%–12% Bis-Tris Gel (Life Technologies, Carlsbad, CA, USA) and transferred to a polyvinylidene difluoride membrane Immobilon-P (Millipore, Bedford, MA, USA) following the manufacurer’s protocol. The membrane was blocked for 1 h with 50 mg/mL skimmed milk in phosphate-buffered saline (PBS) and then incubated with anti-DNAJB3 antibody (Proteintech Group, Chicago, IL, USA) diluted with PBS (1:1000) as a primary antibody overnight. The membrane was washed with PBS three times and incubated with horseradish peroxidase (HRP)-labeled secondary antibody diluted with PBS (1:10,000) for 1 h. The bands were detected using Chemi-Lumi One L Western-blotting detection reagents (Nacalai Tesque, Kyoto, Japan).

### 4.5. Data Analysis

Data were presented as means ± S.D. and were assessed for statistical significance using the unpaired *t*-test. The correlation analysis was preformed using the Spearman rank method. A value of *p* < 0.05 was considered statistically significant.
